# An evaluation of a breastfeeding peer support service in North East England: A qualitative study with service users, practitioners and volunteers

**DOI:** 10.1371/journal.pone.0354027

**Published:** 2026-07-27

**Authors:** Kerry Brennan-Tovey, Simon Barrett, Louise Hayes, Malcolm Moffat, Gina Nguyen, Maria Raisa Jessica (Ryc) Aquino, Judith Rankin

**Affiliations:** Population Health Sciences Institute, Faculty of Medical Sciences, Newcastle University, Newcastle upon Tyne, United Kingdom; University of Cambridge, UNITED KINGDOM OF GREAT BRITAIN AND NORTHERN IRELAND

## Abstract

**Background:**

Breastfeeding has health benefits for women and infants. Although the WHO recommends that infants are breastfed exclusively for the first six months, exclusive breastfeeding rates remain low in the UK, with many women stopping breastfeeding within six weeks of their infant’s birth. Many women face challenges initiating and continuing breastfeeding, including low confidence, limited support and misinformation. Breastfeeding peer support (BPS) services play a vital role in supporting women to continue to breastfeed by connecting new mothers with trained volunteers who offer advice, emotional encouragement and reliable information.

**Methods:**

This study aimed to undertake a formative evaluation of the BPS service in Newcastle upon Tyne from the perspective of those who deliver the services, the wider healthcare team and mothers’ experiences of utilising the service. This evaluation used a qualitative design, consisting of one-to-one semi-structured interviews with practitioners, volunteers, healthcare professionals and service users. Recruitment took place between 28^th^ March – 14^th^ May 2025. Recorded interview data were transcribed verbatim and analysed using Braun and Clarke’s coding reliability thematic analysis.

**Results:**

Twenty-two interviews were conducted (n = 11 practitioners, volunteers and healthcare professionals and n = 11 service users). Analysis identified four themes: mechanisms of the service; support offered by the service; outcomes of the service, and challenges to service delivery. Overall, the BPS was considered a supportive service by participants.

**Conclusions:**

The normalisation of breastfeeding was seen as crucial to the service, while service users described that the emotional support provided helped to increase their confidence in breastfeeding. Challenges were identified for the service, including engaging those aged under 25 years and those for whom English is a second language. These findings will be used to inform the future development of the service.

## Introduction

Breastfeeding brings benefits to both mothers and infants [1,2]. Although the World Health Organization (WHO) recommends exclusive breastfeeding for six months [3], rates of initiation and continuation remain low, particularly in many high-income settings [4,5]. In England, around 73–74% [6] of women start breastfeeding, but this falls to 55.6% at 6–8 weeks and to around 1% at six months, with the lowest prevalence at 6–8 weeks observed in the North East of England (25.9%) [7]. Breastfeeding peer support (BPS) initiatives aim to connect expectant and new mothers with trained peers with lived experience of breastfeeding, complementing professional care provided by midwives and health visitors. BPS is recommended internationally to improve outcomes and reduce inequalities [8]. The WHO’s Global Strategy [3] and National Institute for Health and Care Excellence (NICE) guidance [9,10] highlight the value of peer counsellors and mother-to-mother groups, in collaboration with healthcare professionals. Peer support provides emotional, practical, and informational help from those with lived experience [11,12].

Across international literature, some trials of peer support programmes have found no effect on increasing breastfeeding rates [13], while others have reported improved breastfeeding rates at six months after birth [14,15]. Despite this uncertainty, and recognised challenges such as funding and professional tensions [16], BPS services are commissioned in 56% of NHS areas across the UK [17].

In Newcastle upon Tyne, a BPS service commissioned by Newcastle City Council (NCC) and delivered by a well-established voluntary and community sector (VCSE) organisation has been operating since 2012. The VCSE organisation routinely collects data from service users including (but not limited to) postcode, ethnicity, age of mother and age of infant. Initially the programme targeted postnatal support for families in socioeconomically deprived areas. It has recently expanded – through funding from the national *Start for Life* initiative – to incorporate antenatal provision designed to enhance both breastfeeding initiation and continuation rates.

The service offers structured practical and emotional support during pregnancy and the postnatal period. Practitioners are trained to undertake breastfeeding assessments as well as to provide emotional support and facilitate groups. These practitioners also receive ongoing training and education which follows UNICEF guidelines. Antenatal support includes preparation for childbirth, expectations during labour, and infant feeding education, delivered via telephone consultations and community-based group sessions held four times a week. Postnatal support is offered to mothers who express an intention to breastfeed, with contact initiated within 48 hours of hospital discharge. Women residing in areas of higher deprivation receive a telephone call, while those in less deprived areas are contacted by text message. Support for breastfeeding mothers is provided either one-to-one or in group settings by trained VCSE practitioners. Peer volunteers who themselves receive 12 weeks of training in various aspects of breastfeeding also help to support the service in various ways, by attending groups or facilitating online activity for example.

Volunteers, practitioners and healthcare professionals have different roles within the organisation. Volunteers are mothers who are either a current or former service users who have breastfed their baby. They undergo brief training on how to provide peer support. Practitioners are employed full-time by the voluntary organisation to provide support to pregnant women and breastfeeding mothers. They have lived experience of breastfeeding themselves and have undergone more intensive training. Finally, healthcare professionals, are professionals who have been educated to at least degree level and are registered with the appropriate governing body (e.g., midwife and/or health visitor).

This study aimed to undertake a formative evaluation of the BPS service in Newcastle upon Tyne from the perspective of those who deliver the service, the wider healthcare team and mothers’ experiences of utilising the service. The evaluation was undertaken to provide: an independent perspective of the service, insights into the barriers to service delivery, and recommendations for service redesign to NCC to inform its recommissioning.

## Methods

### Design

A formative qualitative evaluation of the BPS service, consisting of one-to-one semi-structured interviews with practitioners and volunteers delivering the service, members of the wider healthcare team and service users.

### Participant recruitment and sampling

Participants were recruited to take part in a single one-to-one semi-structured interview (via MS Teams or telephone). Purposeful sampling [18] with snowballing sampling [19] was used following the eligibility criteria (see Table 1) with service users having to meet all the inclusion criteria, and practitioners were required to meet one. Recruitment took place between 28^th^ March – 14^th^ May 2025.

**Table 1:** Study inclusion criteria.

Service users	Practitioners/volunteers/healthcare professional
Mothers (and partners) who have accessed the service a minimum of two times	Professional/volunteer staff who deliver the service within the VCSE organisation
Mothers (and partners) who have accessed the service within the past 18-months	Managers who operate the service within the VCSE organisation
Mothers (and partners) living in Newcastle at time of interview	Commissioners who fund the service
	Healthcare professionals who interact with the service (i.e., Midwives, Health Visitors and family hub staff)

Recruitment was via KBT attending the BPS social groups and speaking with service users to determine if they met the eligibility criteria before inviting them to participate in the study; practitioners sharing study materials with service users who met the inclusion criteria; recruitment posters and flyers shared in breastfeeding settings, and emails to practitioners, healthcare professionals and volunteers.

Participation was entirely voluntary. Participants were provided with a participant information sheet and a consent form and given the opportunity to ask questions and receive satisfactory responses. Written or verbal consent was obtained prior to participation.

### Data collection

Interviews were conducted by KBT and SB via telephone or MS Teams and were recorded with consent. Interviews lasted between 18 and 51 minutes using two bespoke topic guides, one for professionals and one for service users (S1 File). These were informed by existing literature [20–23], input from the research team, key stakeholders and a specialist breastfeeding practitioner.

Broad interview topics for service users included history and background of breastfeeding, experiences of accessing the BPS service, perceived benefits of engagement, and recommendations for future services. For practitioners/volunteers/healthcare professionals, topics included their role and involvement, knowledge of, experiences and attitudes of the BPS service, perceived impact the BPS has on mothers, families and services, and recommendations for future service developments. Service users were provided with a £20 Love2Shop voucher to thank them for their time.

Data were collected until KBT and SB agreed that no new information was being obtained from the interview data [24].

### Data analysis

Interview data were transcribed verbatim by an external transcription company and anonymised by KBT and SB before being imported into NVivo 15 [25]. An inductive coding reliability thematic analysis [26–29] was undertaken, to allow reliability in coding between all authors.

Anonymised transcripts were thematically coded by KBT, SB, MRJA, MM, GN, LH and JR. Initially, four transcripts (practitioners/volunteers/healthcare professionals (n = 2), and service users (n = 2)) were coded independently by KBT and SB, and subsequent discussions resulted in two codebooks being developed. The remaining transcripts (n = 18) were independently coded by KBT (n = 8), MRJA (n = 2), MM (n = 2), GN (n = 2), LH (n = 2) and JR (n = 2) utilising the codebooks developed. KBT provided coders with the codebooks and code definitions. After coders had completed their individual coding, KBT had one-to-one meetings with all coders, where discussions about the codes, the codebook and to review the coded data took place. Any disagreements were discussed in these meetings and resolved. The two fully coded datasets were independently reviewed by KBT and SB, who then met to discuss the coded datasets and develop preliminary themes. Themes were developed by KBT and SB with input from MRJA, MM, GN, LH and JR.

### Ethical considerations

Ethical approval for this study was granted by Newcastle University Faculty of Medical Sciences Ethics Committee [55859/2023] on 7^th^ February 2025.

## Results

We undertook 22 one-to-one semi-structured interviews with practitioners, volunteers and healthcare professionals (n = 11), and service users (n = 11).

A breakdown of demographics of practitioner, volunteers and healthcare professionals including gender, ethnicity, organisation, and role, is provided in Table 2, and demographics of service users including age, ethnicity and level of support received, is presented in Table 3.

**Table 2:** Practitioner, volunteer and healthcare professional demographic information.

Demographic		N = 11 (%)
Gender	Female	11 (100%)
Ethnicity	White British	11 (100%)
Organisation	NHS	2 (18.2%)
	VCSE organisation delivering service	9 (81.8%)
Role	Clinical Infant Feeding practitioner	2 (18.2%)
	BPS Practitioner	4 (36.3%)
	Volunteer	3 (27.3%)
	Manager	2 (18.2%)

**Table 3:** Service user demographic information.

Demographic		N = 11 (%)
Age range (years)	25-29	1 (9.1%)
	30-39	9 (81.8%)
	40-49	1 (9.1%)
Ethnicity	White British	9 (81.8%)
	White Scottish	1 (9.1%)
	White other	1 (9.1%)
Parent	Mother	10 (90.9%)
	Partner	1 (9.1%)
Number of children	1	8 (72.7%)
	2	1 (9.1%)
	3	2 (18.2%)
Breastfed older children	Yes – all children	0
	Yes – some children	1 (9.1%)
	No – none of their previous children	2 (18.2%)
	Not applicable (do not have older children)	8 (72.7%)
Level of support received	Group setting only	2 (18.2%)
	Telephone only	0
	Both	9 (81.8%)

Coding reliability thematic analysis [27–29] resulted in four overarching themes, and two subthemes: mechanisms of the service (normalisation of breastfeeding and peer experience and the cycle of support); support offered by the service; impacts of the BPS service; and challenges of service delivery. A breakdown of themes, subthemes and descriptions are presented in Table 4. Illustrative quotes are provided for each theme. Participants were allocated a participant number, e.g., PR001 (practitioner), VOL002 (volunteer) or SU003 (service user).

**Table 4:** Themes, subthemes and description.

Theme	Subtheme	Description
1. Mechanisms of the service	Normalisation of breastfeeding	This subtheme explores the normalisation of breastfeeding which was identified as a central mechanism underpinning the service, facilitated within group settings, allowing for an environment that validates both the challenges and successes of breastfeeding.
	Peer experience and the cycle of support	This subtheme explores how practitioners and volunteers have experience of breastfeeding and often have previously accessed the support offered as service users, before becoming volunteers themselves through training delivered by the service.
2. Support offered by the service	–	This theme explores and highlights the emotional and social support offered by practitioners, volunteers and other service users that extends to outside the social groups.
3. Impact of the breastfeeding peer support service	–	This theme explores and highlights the impact the BPS has on both the practitioners, volunteers and service users; this included, for example, service users reporting an increased confidence in their ability to breastfeed, while some volunteers receive additional training to qualify as midwives.
4. Challenges of service delivery	–	This theme explores the challenges of service delivery including travel barriers for service users and ensuring women living in socioeconomically deprived areas and where English is not a first language are engaged and supported through the service.

### Theme 1: Mechanisms of the service

#### Normalisation of breastfeeding.

Practitioners and volunteers identified the normalisation of breastfeeding as a central mechanism underpinning the service. This was predominantly facilitated within group settings, which were described as fostering “a community within a community” where breastfeeding was “normalised and celebrated.” One practitioner explained,

“So, it really creates this kind of culture, or community within a community, where breastfeeding is normalised and celebrated, where mums can come. They can talk to other people who just understand what they’re going through, understand breastfeeding behaviour, and just feel like a safe space where their views are going to be respected and supported.” (PR004)

This process was viewed as particularly important in areas with low breastfeeding prevalence and limited experience within families where*, “well-meaning advice such as to ‘give babies a bottle if they are hungry again’ may undermine breastfeeding journeys”* (PR004).

As one volunteer noted, *“a lot of women come in because they’re having that noise from family... when you’re there and you’re seeing other mams [breast]feeding just as often as you, it helps to normalise it”* (VOL002)

However, several practitioners raised concerns that some mothers felt reluctant to attend groups until they were “fixed”, *“I think sometimes they have this perception that there’s going to be all these women in a circle feeding beautifully, and they don’t fit into that circle… It’s, ‘Maybe in a few weeks.’ It’s like, ‘You need it [support] now.’”* (PR001)

Maintaining an environment that validates both the challenges and successes of breastfeeding was therefore viewed as essential to fostering inclusivity and sustaining engagement.

#### Peer experience and the cycle of support.

Another key mechanism underpinning the service related to its delivery by practitioners and volunteers who possess both personal experience of breastfeeding and a strong commitment to promoting it. Importantly, most staff and volunteers had previously engaged with the BPS service as service users, creating a “cycle of support.” As one practitioner explained,

“I think it’s really important, with it being a peer support team, that we all have absolutely been there, and we’ve been there throughout the journey. We’ve been mums. We’ve then volunteered… we’ve done the full scale of the project.” (PR004)

This cycle of support was maintained through the identification of potential volunteers from among current group participants, *“I would say the majority of the recruitment comes from women who are already accessing our service...It’s often people who have overcome a difficulty or just show a real passion.”* (PR004)

Following recruitment, prospective volunteers meet with the volunteer coordinator, who assesses suitability and obtains references. Training spans 12 weeks and, while structured, is delivered in an informal, family-friendly environment. Volunteers described how training enhanced their knowledge, confidence, and their own personal breastfeeding journeys, *“Yeah, [the training] was quite full-on, but I enjoyed it at the same time; it was interesting just to learn more about breastfeeding and what I was going through myself.” (VOL002).* However, reliance on volunteers makes retention a challenge, particularly as many eventually return to work or other commitments, *“It’s great when everyone’s on maternity leave… But everyone goes back to work, and any sort of volunteering always drops to the bottom of the list. So, it’s the constant recruitment and renewing of volunteers, as well.”* (PR003)

The peer-led model was a strength and a challenge for service sustainability. The recruitment of former service users fosters authenticity, empathy, and trust, and provides continuity and a sense of community. However, the cyclical nature of volunteer turnover highlights the inherent tension in volunteer-dependent service models, where sustaining a “cycle of support” requires ongoing investment in volunteer recognition, development, and coordination.

### Theme 2: Support offered by the service

Service users reported that the community social groups were a great place to receive social and emotional support from practitioners, volunteers and other mothers.

Some mothers reported that the groups were welcoming and friendly. Mothers reported that they felt supported in the groups, and were reassured that all those attending had lived experience, and they would not experience judgement,

“Because sometimes it can be a bit overwhelming if, say, the baby’s crying or whatever and they’re not latching and there’s a whole room of people, but you know that they’re not judging or anything like that, but sometimes I think it’s the extra pressure that maybe that person doesn’t need.” (SU010)

Other mothers reported that they felt the groups were cliquey to begin with, which left them feeling isolated. However, it was reported that this improved over time, otherwise they would not have continued attending, *“Yeah. But I think that particular group, where I thought there was a clique, the second time seeing those mums was two months after, just because everybody goes everywhere, and then I thought they were quite friendly. Although one person was a little bit overbearing.”* (SU009)

Mothers reported that the support offered within the groups extended beyond these settings, for example, through a WhatsApp group where they can speak to each other when in need of support or encouragement,

“Yeah, definitely. I mean, the groups at [AREA IN CITY] has got a WhatsApp chat, but it, kind of, goes a bit quiet from time to time. But it means that if mums are having a really bad night, or they’re wide awake because little one is up, and not wanting to sleep, they can be like, “Hi, really awful night.” And 9 times out of 10, there’s somebody who’s awake.” (SU004)

Mothers reported that they felt the practitioners and peer support staff were very knowledgeable and were trusted with the information they shared. However, mothers reported that they were unaware of other services on offer until their child was older, something they wished had been shared with them earlier on, *“Yeah, I think, obviously, being practitioner-led is a great setup, because they’re the ones that have the knowledge base. And obviously, as peer supporters, they’re parents as well who are going through, or have just gone through, their breastfeeding journey, so can provide that additional support.”* (SU004)

Several mothers reported receiving conflicting advice from practitioners at VCSE organisations and some healthcare professionals, *“From outside of [VCSE organisation] compared to in [VCSE organisation] and at the hospital, [it differed] massively. We’ve stopped going to the [local] clinic because they kept saying you need to stop breastfeeding and giving her more food and things like that and encouraging her to stop.”* (SU011)*.*

Despite the occasional conflicting advice, most mothers described how they felt the information they received from different agencies was coherent. They also acknowledged how the complex nature of breastfeeding, and the need for individually tailored solutions to challenges, meant that any information or advice was likely to vary and be very context dependent.

“I feel like everything’s aligned. I always feel like, it’s not they just tell you, “This is the way to do it.” It’s almost like you’re given a couple of different options as such. It’s almost like you’ve got a bit of homework, “You can try this, this, and this, and if it doesn’t work, do this.”” (SU010)

### Theme 3: Impact of the breastfeeding peer support service

Mothers reported that the support groups allowed for breastfeeding to be normalised, while identifying several further outcomes and practical benefits associated with the service. Mothers reported that the groups allowed them to gain confidence with breastfeeding outside of the home before they started to feed in public,

“Whereas, going to the social group is a really good and helpful halfway house, almost, because it’s like, outside of your set environment, but it’s a safe space where you know that everyone who’s there is on the same page, if that makes sense, and you don’t need to feel like you need to strategically cover up or whatever. And so, you can almost practice breastfeeding in a different environment, but somewhere that’s supportive.” (SU008)

Mothers reported experiencing increased confidence; developing friendships; advice on returning to work; validation of their breastfeeding experiences; education specific to their child (e.g., feeding, development milestone, health and wellbeing); empowerment to make the best decision for them; and encouragement to continue breastfeeding. These were reported as benefits to the support provided by practitioners from VCSE organisations and peer support volunteers,

“the support that [PRACTITIONER] gave was really practical, basically practical pointers for how to do that better, because I think you know the theory of, like, “Oh, and then their chin does this, and then they open their mouth wide, and then whatever,” but then if the baby’s not doing that, you’re, kind of, like, “It’s not lining up with the theory, but I don’t know how to make it better.”” (SU008)

In addition to the practical breastfeeding guidance and the emotional support provided to mothers, practitioners also described positive outcomes for volunteers. Reclaiming parts of their identity and a sense of accomplishment by supporting others were highlighted, *“Because when you become a mum, you’re the baby’s mum, you’re not yourself anymore. But if you’re coming to training every week, and you’re learning something, and you’re being called by your name, it doesn’t sound a lot, but it’s amazing.”* (PR003)

Cascading information and support into the wider community were also described as unanticipated outcomes of the service, *“Some [volunteers] go on to become midwives, a lot of them go on to work in care. We’ve had a lot of trainee GPs, who say they get one day on breastfeeding, they come to us, and then they’re supporting a lot of women more, because of that.”* (PR003)

Volunteers also described how their involvement with the service was driven by a sense of paying back but also brought positive outcomes in terms of employability and confidence,

“I felt really strongly about giving back to the community, the help that I had got. I love being part of my community. I love giving back to the community...You think you’ve done some good in the world. It’s a good thing you could put on your CV, as well. So that would help you with future employment, that kind of thing... a lot of [my] confidence in facilitating groups [at work] came from my role as a volunteer.” (VOL009)

These findings illustrate that the service provides more than breastfeeding education. It is also a space which fosters confidence and empowerment. Moreover, the spillover of knowledge and advocacy into wider social and professional spheres suggests that the service may contribute to changes around breastfeeding practices beyond its immediate participants.

### Theme 4: Challenges of service delivery

Practitioners and volunteers indicated that a significant challenge facing the service was to ensure that it could be delivered to different population groups, particularly those in more deprived areas and those for whom English is not a first language.

Mums aged 25 and under were a target population for increased support for the BPS service but working with these younger parents was described as challenging,

“I think the younger mums are quite difficult to engage. We don’t get many referred in, but we do get some. We often find that they’re quite engaged with us at the start, and that tails off a little bit. So, that’s something that we’re working on at the moment and looking at different ways to engage them, because sometimes, I think, maybe the standard model might not fit them…Sometimes it might need a more tailored approach.” (PR004)

The social groups are targeted at geographical areas where breastfeeding rates are lower and are situated within the more deprived areas of the city. Practitioners described, however, how there were still some issues with access for some families, where mothers who had recently given birth did not feel able to take public transport necessary to attend the groups, *“Mums that live quite far away from some of the groups that we run, and they don’t drive. They don’t have the funds for a taxi. They don’t want to get on public transport when their baby is, like, a week old.”* (PR005). Mothers also identified the distance to travel to the sessions as a barrier if they did not have a car or relied on public transport,

“It’s a little bit awkward for me, if I’m honest. So, the group that I attend, in [AREA OF CITY 2], is the easiest to get to. I have to walk to the Metro stop, then- It’s, like, a 20-minute walk. So, it’s alright, but it’s not super easy. But I don’t think there’s anything that can be done. I was actually quite surprised that there is nothing closer to [AREA OF CITY], because I would have expected there to be something a bit closer. But [AREA OF CITY 2] is accessible, reasonably, by Metro.” (SU009).

Despite the groups being situated in the more deprived areas of the city, the perception among some volunteers and practitioners was that mothers accessing the groups were travelling there from more affluent areas,

“It’s attracting these women from not the target demographic, so they’re coming from further afield to access the support [from more affluent area]. That takes away from that, what I spoke about earlier, about creating that community within a community, because there’s already breastfeeding [in more affluent area], the rates are a lot higher. So, we do get women from [the local area where the group is located], don’t get me wrong, but a lot of the women in that particular group are coming further afield to access the service.” (PR004)

The absence of an interpretation or translation service occasionally created complications, according to practitioners. Efforts were made to have resources available in different languages, and sometimes volunteers who could interpret were found, but the perception was that the language barrier still impacted on access to the service, and as a result the service was not experienced in the same way as it was by those with English as their first language, *“It doesn’t flow like a group would. I feel like the mums then miss out on that network building and that support building, because they’re not able to have these kinds of fluent conversations with other participants”* (PR004)*.* The issue of access and language was also expressed by health professionals, who also identified a lack of representation of Black and minoritised ethnicities among BPS practitioners and volunteers, as well as midwives,

“I think it’s having people that look like yourself. We don’t really have that, from what I can see of the peer support team. It’s our [midwife] team as well... Certainly when I meet people from different cultures what they need from me feels different. When I see them, they need something different. Particularly in the west of the city - we have a really diverse population.” (PR001)

One mother with a disability reported that the room set up prevented her from being able to engage with her daughter effectively in the social group setting, for risk of falling due to numerous trip hazards (e.g., smaller play mats spread across the room),

“It was laid out as lots of big mats in the middle, everyone used to sit around and everyone used to talk together. If you needed a bit of privacy or you wanted to talk to the practitioner separately, you could go off to the side or whatever, but it was very community group-based and, when [PRACTITIONER 4] took over that group, they decided to change how it’s being set up so it’s now three or four small mats. … It’s made it a lot harder accessibility-wise for me.” (SU011)

## Discussion

From interview data we described four main themes: ‘mechanisms of the service’, ‘support offered by the service’, ‘outcomes of the services’ and ‘challenges to service delivery’. Overall, the BPS was considered an important and supportive service by both service users and practitioners and volunteers, with many positive reported outcomes for service users engaging in the service and volunteers delivering the service. The normalisation of breastfeeding was considered to be a vital mechanism of the service and is aided by the delivery of the service by passionate and experienced practitioners who had often previously utilised the service as service users. Previous research [30] found that breastfeeding support provided by trained experienced peers facilitated the normalisation of breastfeeding.

Practitioners noted that the target demographic for the service was women under 25, and those living in the most deprived areas of the city, and while the social groups were situated in deprived areas across the city where breastfeeding rates were lower, this population was not being reached. Practitioners were aware that their current approach may not be suitable for women under 25, and that a tailored approach may be required to engage this population; something they were aware of but not actioning at the time of the evaluation. Service users reported barriers to attending the groups being the cost of travel to and from, with practitioners also noting that those who were attending the groups were those living in more affluent areas of the city and making the longer journey to the groups. Healthcare professionals highlighted that there was a lack of representation of those from Black and minoritised ethnicities amongst healthcare professionals, practitioners and volunteers, something that would be difficult if the service users also lack diversity considering the cycle of support in place within the service. Finally, it was noted by practitioners that there was no language translation services available during the groups for those where English is not a first language, an additional barrier to those living in poverty.

Service users reported that they received social and emotional support from practitioners, peer support volunteers and other mothers attending the groups. While some reported the groups were welcoming and friendly, others felt the groups were ‘cliquey’ and took time to feel welcomed into the community social space. A small group of mothers had developed friendships with other mothers in the groups, which had led to support outside of the group setting and the development of WhatsApp groups. This social aspect has previously been described in research [22]. This sense of community and friendship brings obvious benefits, as well as providing potential barriers to those outside of this community who may feel excluded.

Service users reported that they felt the information being shared with them via practitioners and volunteers was trustworthy. However, a few mothers highlighted that there were times when conflicting information was shared with them from practitioners and volunteers within the service and healthcare professionals outside of the service. Previous research [31], found similar experiences, with breastfeeding mothers reporting receiving conflicting advice, and having to process the confusion this caused. Given the complexity of breastfeeding and the individual nature of issues faced, it is perhaps unsurprising that there is sometimes a sense of being overwhelmed with information being shared by different groups of people with different experiences.

Service users reported many positive outcomes of engaging with the service, including confidence building; feeling empowered in their decision-making regarding infant feeding; validation of their experiences; support specific to their child and encouragement to continue breastfeeding, findings that confirm previous research [16]. Mothers reported that these positive experiences allowed them to continue breastfeeding and they often reflected that without the support of the practitioners and volunteers they would not have continued breastfeeding [32]. Peer support volunteers reported that they felt a sense of achievement in providing their time and experience to other breastfeeding mothers in their capacity as volunteers, with many of the volunteers having previously used the service themselves, they felt it was a way for them to give back to the service and future breastfeeding mothers. Previous studies [22,33] have reported similar findings of peer support volunteers feeling duty-bound to share their own experiences and evidence-based knowledge.

This study provides evidence of the support offered by the BPS to mothers, which included emotional and social support as well as breastfeeding education. This support improved mothers’ confidence and allowed them to be empowered in their decision-making around infant feeding. However, the study also provides evidence of some of the challenges of delivering the service, including ensuring the service is accessible to the those under 25 and those living in poverty, to those where English is a second language, those who rely on public transport, and those with disabilities.

### Service and policy recommendations

From the interview data several service and policy recommendations have been developed including hosting some groups in the evenings and weekends and in areas that are well serviced by public transport. The ability to offer initial appointments one-to-one with a practitioner in the women’s home is recommended as well as ensuring that group settings are accessible to those with physical disabilities. The study team recommends increasing representation from minoritised groups in both practitioners and volunteers to enable culture to be understood and shared, alongside the use of translators and interpreters in services. Finally, we recommend organising groups that are tailored to the under 25s. Actioning these recommendations will help to ensure the service provision is equitable.

### Strengths and limitations

A strength of this evaluation is drawing on multiple perspectives of the service, from providers, users and healthcare professionals who refer to the service. Another strength is the number of researchers who were involved in the analysis of interview data and theme development, ensuring more accurate and comprehensive interpretation of data.

A limitation of the study is the lack of diversity in the participants, with practitioners, volunteers, and healthcare professionals and service users all identifying as White. This aligns with the challenge identified in the study of engaging diverse populations into the service. A final limitation of this evaluation was the missed perspectives of those who declined to engage with the service.

## Conclusion

This study has demonstrated that the BPS is an important and supportive service with reported positive outcomes by service users, including normalisation of breastfeeding, social and emotional support and friendship building. However, the study has demonstrated that there are still barriers to engagement by those from minoritised groups. Recommendations have been provided to help shape and redesign BPS to ensure the service is equitable to all breastfeeding mothers.

## Supporting information

S1 File Topic Guides. The two topic guides used to generate interview data.
(DOCX)
