# Supplementary material for: An evaluation of a breastfeeding peer support service in North East England: A qualitative study with service users, practitioners and volunteers
Source: PLoS One. 2026 Jul 27;21(7):e0354027. doi: 10.1371/journal.pone.0354027 (PMC13405085; doi:10.1371/journal.pone.0354027)
Supplement: S1 File — The two topic guides used to generate interview data. (DOCX) [file pone.0354027.s001.docx]

**Supplementary File 01. Topic Guides**

Evaluation of a breastfeeding peer support services in Newcastle

Interview topic guide – Service users

Researcher introduces themselves and reminds participant of confidentiality.

Thank you for agreeing to take part in this interview, and for giving up your time. We are inviting women and their partners who have used or are using the breastfeeding peer support services in Newcastle to help us understand your experiences of using the service, and what recommendations could be offered to improve the service.

The interview will last no longer than an hour and there will be an opportunity for you to provide any further information that has not been covered. Let me know if you would like to have a break or stop the interview at any point. Feel free to ask questions at any stage during the interview.

**History and background**

*These first questions are quite general and are about you and your family, and where you live.*

Can you tell me a little bit about you and your family? (e.g., how many children do you have? How old are they? Where do you live? Do you have a partner?)

What do you and/or your partner do for work?

Tell me about your experience with breastfeeding with this birth, and any previous births

What information or education did you receive during your pregnancy about breastfeeding?

Where did you receive that information/education?

The focus of today is your experiences of using the peer support breastfeeding service in Newcastle.

**Experiences of accessing the BPS**

How did you hear about the service?

Who were the professionals who told you/referred you to the service? (how did they tell you about the service?)

How long did you wait between being referred and being seen by the services?

What has been your involvement with the service? (i.e., when was your first contact made and how? How frequent was the contacts? Where did the contacts take place? Etc) – (probe into time of appointments, travel requirements, etc.).

Who were the professionals whom you have met through this service? (professionals, peer support staff, volunteers etc.).

What other advice/support around infant feeding have you received? Has this been from healthcare professionals? (who?), family? Social media?

What types of support did you receive? (i.e., infant feeding, emotional support, help with ‘other’ issues, advice regarding the potential contraceptive effect of breastfeeding)How long did you receive support for? Where you discharged, or did you feel you didn’t require any additional support?

What do you think of the support you received?

Do you think/did you feel you received any conflicting advice around breastfeeding?

Enablers – what worked well? What did you like?

Barriers – what didn’t work well? What didn’t you like?

**Benefits of engagement**

What have been the benefits (if any) of receiving support?

(explore in relation to infant feeding, maternal wellbeing, parenting experiences, meeting new people/social opportunities etc.)

What has been the impact of this support on your infant feeding experiences?

**Recommendations/changes to practice**

Do there need to be any changes or developments to how the service is offered or provided?

(explore the answer –thinking methods of engaging with the service, time of day of services, communication methods, provision of services etc)

**Any further information**

Anything else that you would like to add that has not already been discussed?

Do you have any questions?

Remind the participant about confidentiality.

Ask for their address (if remote interview) to post £20 Love2shop voucher to them. If in person – provide them with voucher

Thank you for your time and involvement.

Evaluation of a breastfeeding peer support services in Newcastle

Interview topic guide - Professionals

Researcher introduces themselves and reminds participant of confidentiality.

Thank you for agreeing to take part in this interview, and for giving up your time. We are inviting a number of healthcare/community/voluntary group professionals from Newcastle to take part, so I am pleased that we are meeting today.

We are interested in finding out about your experiences of delivering/engaging/referring to the peer support breastfeeding services in Newcastle, to better understand how these services operate, and what recommendations could be offered to improve the service for the women utilising the service, the professionals and peer support staff delivering the service and those professionals who engage with the service.

The interview will last no longer than an hour and there will be an opportunity for you to provide any further information that has not been covered. Let me know if you would like to have a break or stop the interview at any point. Feel free to ask questions at any stage during the interview.

**Your role and involvement**

Role: Could you tell me about your current occupation and responsibilities?

Setting: Could you provide some information about the setting that you work within (i.e., NHS/healthcare/community)

Involvement: In what ways are you involved in supporting breastfeeding women, or women who wish to breastfeed?

In what way are you involved in the BPS service?

**Knowledge of, experience and attitudes towards BPS**

What do you know about the service? (where did you get the information about the service? Or what does the service look like? (can you explain it to me))

How have you been involved in the service?

What has been your experience of the service?

How is the service working within practice?

Enablers – what is working well? Why?

Barriers – what is not working so well? Why?

How are peer supporters engaging with women? (any issues of non-engaging with certain women/population groups?)

Have there been any developments/changes to how the service is provided?

Do you feel the service is embedded within the wider healthcare services provided to women?

**Impact of service on women, families and professionals**

What (are your perceptions of the) support being provided to women?

What do you think are the benefits of the services for women and/or families?

What impact do you think the service has had on infant feeding practices in the North East, maternal wellbeing, parenting experiences?

What, if any, are the benefits to the peer supporters and/or the wider healthcare professionals (i.e., midwives, health visitors, family hub workers etc)?

Have there been any wider benefits of the service? (i.e., in terms of how the service is provided, training opportunities for staff, women forming networks/peer support, changing cultures around breastfeeding, reducing workload for healthcare professionals).

**Recommendations/changes to practice**

Do there need to be any changes or developments to how the service is offered or provided?

(explore the answer –thinking training, access, referral process, methods of engaging women (especially those from high levels of deprivation), communication, provision of services etc.)

**Any further information**

Anything else that you would like to add that has not already been discussed?

Do you have any questions?

Remind the participant about confidentiality

Thank you for your time and involvement.
