# Supplementary material for: An evaluation of a breastfeeding peer support service in North East England: A qualitative study with service users, practitioners and volunteers
Source: PLoS One. 2026 Jul 27;21(7):e0354027. doi: 10.1371/journal.pone.0354027 (PMC13405085; doi:10.1371/journal.pone.0354027)
Supplement: Attachment — Submitted filename: response_to_reviewers_2026_05_18.docx [file pone.0354027.s003.docx]

An evaluation of a breastfeeding peer support service in North East England: a qualitative study with service users, practitioners and volunteers

Dear Maria Berghs,

Thank you for sharing the below feedback from two independent reviewers. After actioning the comments to the manuscript, I would like to re-submit the manuscript titled ‘An evaluation of a breastfeeding peer support service in North East England: a qualitative study with service users, practitioners and volunteers’ to PLOS One. Below is a table outlining all the feedback received and out responses to these comments.

Best wishes,

Dr Kerry Brennan-Tovey on behalf of the study team

**Editor**:

| Comment | Response |
| --- | --- |
| Please ensure that your manuscript meets PLOS ONE's style requirements, including those for file naming. The PLOS ONE style templates can be found at  <https://journals.plos.org/plosone/s/file?id=wjVg/PLOSOne_formatting_sample_main_body.pdf> and  <https://journals.plos.org/plosone/s/file?id=ba62/PLOSOne_formatting_sample_title_authors_affiliations.pdf> | Thank you for this comment. We have made sure that all files are formatted and have been named according to the templates. |
| Please include captions for your Supporting Information files at the end of your manuscript, and update any in-text citations to match accordingly. Please see our Supporting Information guidelines for more information: <http://journals.plos.org/plosone/s/supporting-information>. | Thank you for this comment. This has been actioned. |
| We note that you have indicated that there are restrictions to data sharing for this study. For studies involving human research participant data or other sensitive data, we encourage authors to share de-identified or anonymized data. However, when data cannot be publicly shared for ethical reasons, we allow authors to make their data sets available upon request. For information on unacceptable data access restrictions, please see <http://journals.plos.org/plosone/s/data-availability#loc-unacceptable-data-access-restrictions>.  Before we proceed with your manuscript, please address the following prompts:  a) If there are ethical or legal restrictions on sharing a de-identified data set, please explain them in detail (e.g., data contain potentially identifying or sensitive patient information, data are owned by a third-party organization, etc.) and who has imposed them (e.g., a Research Ethics Committee or Institutional Review Board, etc.). Please also provide contact information for a data access committee, ethics committee, or other institutional body to which data requests may be sent.  b) If there are no restrictions, please upload the minimal anonymized data set necessary to replicate your study findings to a stable, public repository and provide us with the relevant URLs, DOIs, or accession numbers. Please see <http://www.bmj.com/content/340/bmj.c181.long> for guidelines on how to de-identify and prepare clinical data for publication. For a list of recommended repositories, please see <https://journals.plos.org/plosone/s/recommended-repositories>. You also have the option of uploading the data as Supporting Information files, but we would recommend depositing data directly to a data repository if possible.  Please update your Data Availability statement in the submission form accordingly. | Thank you for this comment and for our email communications regarding this.  De-identified data contains potentially identifying and sensitive information to the participants. Participants were made aware in the participant information sheet how their data will be stored and shared:  *‘At the end of the study, confidential data (for example your name, contact details and audio-recorded interview) will be destroyed. Your anonymised research data will be separated from all your personal details and stored on a secure server within Newcastle University for 10 years in line with Newcastle University Research Data Management Policy Principles and Code of Good Practice.’*  In the consent form participants agreed:  *‘I consent to my anonymised research data being stored and used by others for future research.’*  However, while participants have consented to their data being stored and used by others for future research, they did not consent to their data being stored in a public database, but rather Newcastle University secure servers.  Newcastle University Research Ethics Committee provided ethical approval for this project (Ref: 55859/2023) (incl. participant information sheet and consent form).  A named contact has been identified for all data requests to be submitted to: [maternalandchildhealth@newcastle.ac.uk](mailto:maternalandchildhealth@newcastle.ac.uk)  The data availability statement within the manuscript has been updated to reflect these changes. |
| In the online submission form you indicate that your data is not available for proprietary reasons and have provided a contact point for accessing this data. Please note that your current contact point is a co-author on this manuscript. According to our Data Policy, the contact point must not be an author on the manuscript and must be an institutional contact, ideally not an individual. Please revise your data statement to a non-author institutional point of contact, such as a data access or ethics committee, and send this to us via return email. Please also include contact information for the third party organization, and please include the full citation of where the data can be found.  If the reviewer comments include a recommendation to cite specific previously published works, please review and evaluate these publications to determine whether they are relevant and should be cited. There is no requirement to cite these works unless the editor has indicated otherwise.   Please review your reference list to ensure that it is complete and correct. If you have cited papers that have been retracted, please include the rationale for doing so in the manuscript text, or remove these references and replace them with relevant current references. Any changes to the reference list should be mentioned in the rebuttal letter that accompanies your revised manuscript. If you need to cite a retracted article, indicate the article’s retracted status in the References list and also include a citation and full reference for the retraction notice. | Thank you for this comment. We have identified an institutional point of contact for data requests to be sent to:  [maternalandchildhealth@newcastle.ac.uk](mailto:maternalandchildhealth@newcastle.ac.uk) |
| The reviewers are largely supportive and have requested minor revisions. Do pay attention to those comments about ensuring more critical analysis of the methods and discussion. I would consider the implications on equity of service provision. Do please also check formatting, quotes from your participants and proofread the entire paper. | Thank you for this comment. Particular focus has been made to action all the comments and feedback below.  The authors have also ensured that the manuscript is formatted to PLOS One’s standards. The authors have proofread the manuscript. |

**Reviewer one**:

| Comment | Response |
| --- | --- |
| Each section of the manuscript is well written, and conclusions drawn are appropriate from the results presented. | Thank you for this great comment. |
| lines 130-13 -Can you include more details about how authors were able to determine point of saturation of responses since the carried out one on one interviews? | Thank you for this comment. We have clarified this within the manuscript. |
| line 142-143 should come before lines 140-141 | Thank you for this comment. This section has been edited following reviewer feedback. |
| kindly provide more details about how thematic analysis was carried out to ensure interobserver agreement during coding which is the main advantage of Braun and Clarks coding relativity thematic analysis | Thank you for this comment. The authors have added detail to the data analysis section to clarify how the coders ensured intercoder reliability and how disagreements were resolved. |
| line 163 participants were allocated participant numbers | Thank you for this comment. We have since changed the wording of this sentence. |

**Reviewer two**:

| Comment | Response |
| --- | --- |
| Thank you for this paper. It is a well written paper evaluating the peer support programme from a multiple sources. There are a few minor points to address, mainly to ensure clarity and add context for the readers. | Thank you for your positive comments. We have actioned all the points you have listed below. |
| It is not clear what's the difference between a "practioner" and a "volunteer" or "healthcare professional". It would be good if each term can be clearly defined in the background | Thank you for this comment. And we agree, this distinction is needed. We have added a paragraph within the background highlighting the role descriptions and level of training/education of volunteer, practitioner and healthcare professional. |
| While the objective of the study is clear, the rationale behind the objective isn't as clear. The authors could add more context why the evaluation is needed. The main question I had at the end was "how are these findings going to help the programme?" What changes need to be made? | Thank you for this comment. The evaluation was undertaken independently to provide insight into the service, the barriers of the service, and provide recommendations to the local authority to allow them to redesign the service as it was being recommissioned. The authors have added a brief sentence outlining this within the manuscript alongside the aim and objective of the study. |
| Page 26, Line 9: In the discussion, it was highlighted that one of the limitations was language barrier. In this specific line, being of "black" descent was placed closely with language. I am not really comprehending why that is so. I had thought black women also spoke English | Thank you for this comment. This was written as two separate points, there was no link between the two intended, as you are correct, Black women often speak English. However, we have rearranged the end of this paragraph to show that they are two separate points – 1. That a lack of diversity in volunteers etc is difficult to change if the service users lack diversity (the pool from which volunteers are recruited from); 2. That there lacked interpretation services within the groups for women where English is not a first language. |
| Service and policy recommendations: The recommendation of increasing representation of minority ethnic groups should be more important than translators. Because the support isn't just understanding the language alone but having someone who understands your culture is also important (and that would be lost in translation). | Thank you for this comment. We agree that this needs to be reordered to highlight the importance of understanding and sharing culture. So, we have rearranged that sentence, to place that ahead of the use of translators and interpreters. |
| At the end of the manuscript, a conclusion section is missing. This should be added. | Thank you for this comment. The authors have added a short concluding paragraph at the end of the manuscript. |
